# Multifaceted roles of Epac signaling in renal functions

**DOI:** 10.1042/BCJ20253103

**Published:** 2025-05-14

**Authors:** Oleh Pochynyuk, Kyrylo Pyrshev, Xiaodong Cheng

**Affiliations:** 1 Department of Integrative Biology and Pharmacology, McGovern Medical School, The University of Texas Health Science Center at Houston, Houston, TX 77030, U.S.A.; 2 Texas Therapeutics Institute, Institute of Molecular Medicine, McGovern Medical School, The University of Texas Health Science Center at Houston, Houston, TX 77030, U.S.A.

**Keywords:** aquaporin (AQP), electrolyte transport, epithelial Na^+^channel (ENaC), Na^+^/H^+^exchanger type 3 (NHE-3), polyuria

## Abstract

3',5'-cyclic adenosine monophosphate (cAMP) is a fundamental secondary messenger capable of rapidly amplifying and propagating cellular signals in response to various extracellular stimuli. cAMP plays a significant role in hormone-mediated regulation of renal fluid and electrolyte balance. Impaired signaling of cAMP has been linked to a variety of pathological ramifications in the kidneys. This review explores the physiological functions of exchange proteins directly activated by cAMP (Epac) in renal water balance and the regulation of solute transport in the renal tubule. Additionally, the involvement of Epac signaling in renal pathologies such as acute kidney injury, chronic kidney disease, and polycystic kidney disease is discussed.

## Introduction

Cyclic adenosine monophosphate (cAMP) is a crucial secondary messenger that controls a variety of cellular functions, including metabolism, transcription, apoptosis, cellular trafficking, cell differentiation, and proliferation. cAMP is involved in various cellular processes and is essential for hormone-regulated activities in the kidney, particularly in the collecting duct, where it promotes water reabsorption through arginine vasopressin (AVP) signaling, which is critical for maintaining water balance and urine concentration [1]. Dysfunction in the cAMP signaling pathway has been linked to various kidney diseases [2]. A recent study suggests that urine cAMP is a biomarker to assess the residual function of the collecting duct in the kidney and potentially serves as a prognostic indicator in patients with chronic kidney disease (CKD) [3].

**Figure 1:**
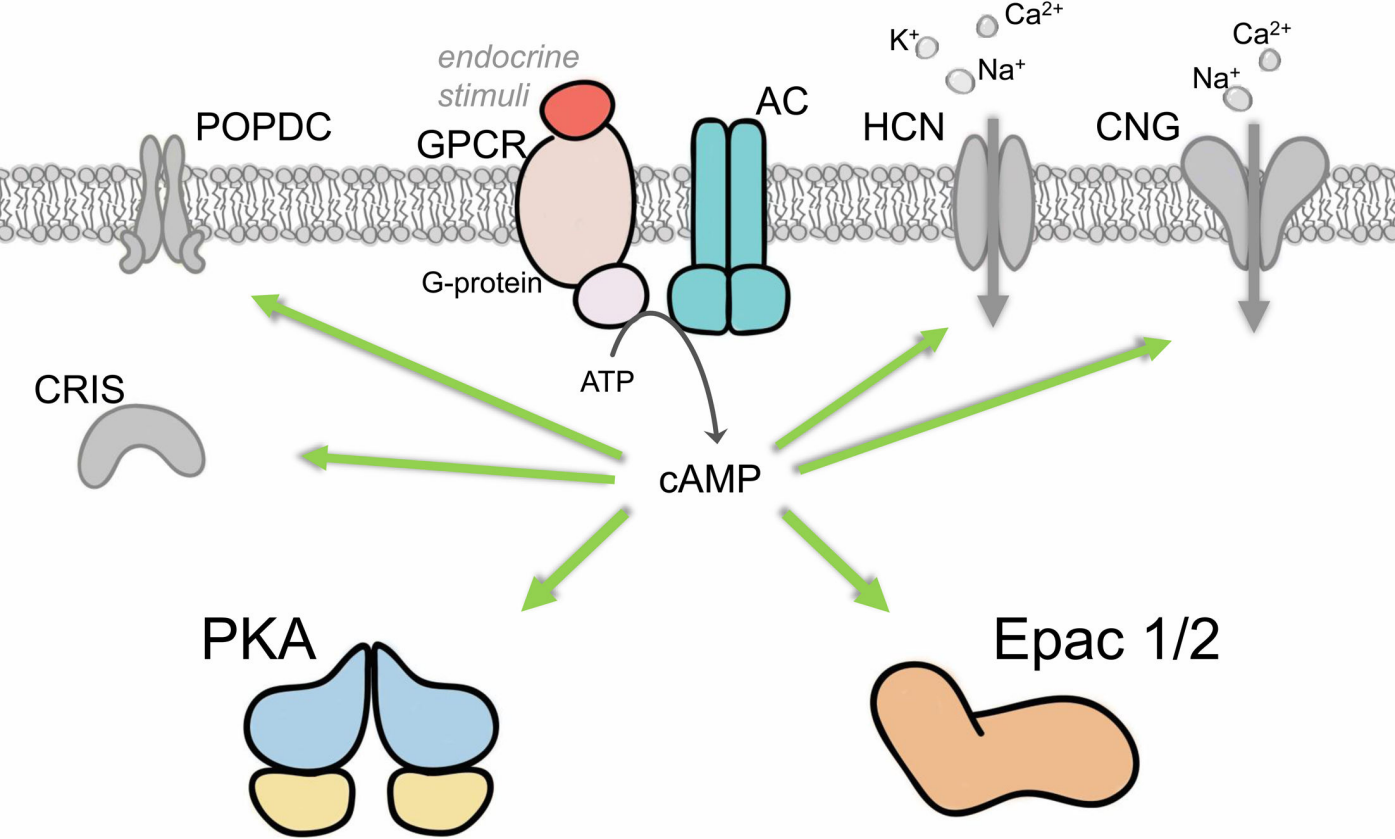
Mammalian cyclic AMP signaling pathways. Activation of the G-protein-coupled receptor (GPCR), and adenylyl cyclase (AC) cascade at the cell membrane in response to the extracellular stimuli leads to the production of intracellular cAMP and subsequent activation of cAMP effectors, including the ubiquitously expressed protein kinase A (PKA) and exchange protein directly activated by cAMP (Epac), as well as tissue-specific cyclic nucleotide-regulated ion channels (CNG and HCN), the Popeye domain containing proteins, and the cyclic nucleotide receptor involved in sperm function.

Intracellular cAMP is produced from ATP when a hormone binds to its cell surface receptor and activates adenylate cyclase via a Gαs protein. In mammals, at least five families of cAMP effector proteins exist (Figure 1): protein kinase A (PKA) [4], cyclic nucleotide-regulated ion channels (CNG and HCN) [5], exchange proteins directly activated by cAMP (Epac1 and Epac2) [6,7], Popeye domain containing proteins [8], and cyclic nucleotide receptor involved in sperm function [9]. Among these cAMP effectors, PKA is the most well studied and has been suggested to play a crucial role in kidney functions [10]. In addition, extensive studies have provided accumulating evidence of a crucial role of Epac signaling in renal physiology [11,12]. In the following sections, we will discuss how Epac proteins regulate renal fluid and electrolyte balance, as well as speculate on their putative therapeutic value in the treatment of acute and chronic renal diseases.

## Epac isoform expression in the renal tissue

**Figure 2:**
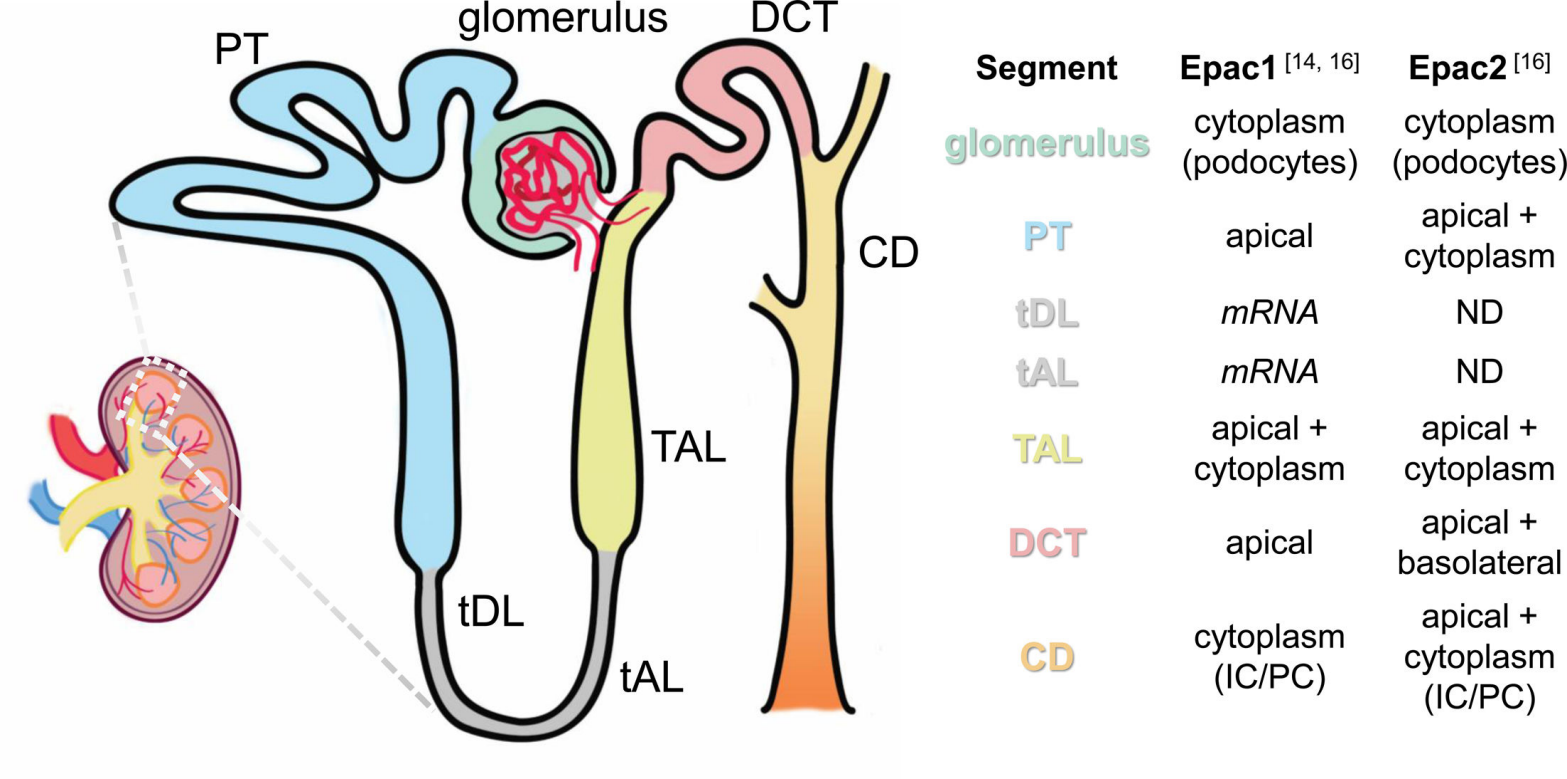
Expression of Epac1 and Epac2 in the nephron. Epac1 and Epac2 transcripts or proteins are detected in various segments of the nephron: CD, collecting duct; DCT, distal convoluting tubule; PT, proximal tubule; ND, not detected; TAL, thick ascending limb; tAL, thin ascending limb; tDL, thin descending limb. Segments without detected Epac protein expression are marked in gray.

Shortly after the discovery of Epac signaling as a physiologically relevant downstream pathway of cAMP [6,7], it was recognized that the kidney has one of the highest Epac expression levels, specifically the Epac1 isoform [7,13], suggesting it may play an essential role in mediating cAMP functions in the kidney (Figure 2). Widespread Epac1 mRNA expression was detected by reverse transcription-PCR analysis of micro-dissected rat nephron segments, specifically in the glomeruli, proximal tubules (PT), both convoluted and straight parts, thin descending limbs of Henle’s loop (tDL), thin ascending limbs of Henle’s loop (tAL), thick ascending limbs of Henle’s loop (TAL), cortical collecting ducts (CCD), outer medullary collecting ducts, and inner medullary collecting ducts with the highest expression observed in the CCD [14]. Follow-up studies documented an abundant presence of Epac1 and Epac2 in all segments of the renal nephron, with the exception of the tDL and tAL, showing no appreciable expression of both isoforms [15,16]. Importantly, the comparison of Epac1 and Epac2 expression patterns revealed striking similarities between rodent and human kidneys [16]. While the initial evidence argued against renal vasculature and glomerulus being sites with apparent Epac presence, relatively weak Epac staining was found in the glomerular visceral epithelium (i.e., podocytes), particularly the Epac2 isoform. In contrast, no staining was found in glomerular or peritubular capillaries [16]. In agreement, another study also revealed Epac expression in visceral glomerular epithelium, but not in the glomerular capillaries [17].

In the PT, the expression of Epac1 and Epac2 was primarily restricted to the brush border along the whole length of the segment, while it was lowest in the last straight part (S3 segment). This pattern indicates an active role of Epac in regulating apical transporters, most likely Na^+^/H^+^ exchanger type 3 (NHE-3, *Slc9A3*), as discussed below. In the TAL, Epac isoforms are expressed on both apical and basolateral sides with a slight bias toward the former [16]. However, there is no experimental evidence in support of Epac regulation of TAL function to date, with the only report demonstrating that cAMP-dependent exocytosis of the major Na^+^, K^+^, and Cl^-^ transporter, NKCC2 (*Slc12A1*) is controlled by PKA but not Epac [18]. A similar pattern of Epac isoform expression was also detected in the distal convoluted tubule [16]. Again, the physiological relevance of Epac signaling at this site remains unknown, with no reports documenting either the regulation of major NaCl and Ca^2+^ transporting systems, namely the thiazide-sensitive Na^+^-Cl^-^ cotransporter (NCC, *Slc12A3*) and Ca^2+^-permeable TRPV5 channel. It should be noted, though, that the levels of phosphorylated (i.e., active) forms of NKCC2 and NCC were drastically elevated in mice lacking Epac1 and Epac2 isoforms [19]. However, it was deduced that this is likely a compensatory event secondary to the diminished solute transport in other tubular segments [19,20].

In contrast with the upstream tubular segments, the expression patterns of Epac1 and Epac2 exhibit a notable separation in the collecting duct system, with Epac1 expression being mostly in intercalated cells, whereas Epac2 was found in both principal (more) and intercalated (less) cells [16]. This implies that different Epac isoforms might be involved in governing adaptations to distinct physiological states regulated by these cell types, including acid–base insults (intercalated cells), renal water conservation, and fine-tuning of Na^+^ and K^+^ balance (principal cells) [21,22]. However, the deletion of either Epac1 or Epac2 produces a very similar salt-wasting phenotype in response to dietary Na^+^ restriction, which was attributable to the diminished Na^+^ reabsorption by principal cells [20]. Additional studies are needed to resolve the intricate roles of Epac1 and Epac2 in collecting duct principal and intercalated cells of the collecting duct.

## Epac signaling in renal water balance

Aquaporins (AQPs) are integral membrane proteins that selectively transfer water across cell membranes [23]. At least seven AQPs are found in the human kidney, playing a vital role in maintaining body water balance [24]. AVP-sensitive AQP2 channel is well studied and expressed in the principal cells of the collecting duct. Increases in plasma osmolarity trigger AVP secretion causing stimulation of the vasopressin receptors type 2 (V2R) on the basolateral membrane of principal cells. This activation promotes transcription, translocation, and fusion of AQP2-containing vesicles to the apical membrane, enabling osmotically driven water reabsorption through AQP2, followed by the constitutively expressed AQP3 and AQP4 on the basolateral membrane [25]. Binding of AVP to V2R initiates a series of cAMP-dependent events, including PKA-mediated phosphorylation of AQP2 at Ser256 to promote its translocation to the apical membrane [26–30], which is likely under the control of cAMP-dependent calcium mobilization from ryanodine stores [31].

**Figure 3:**
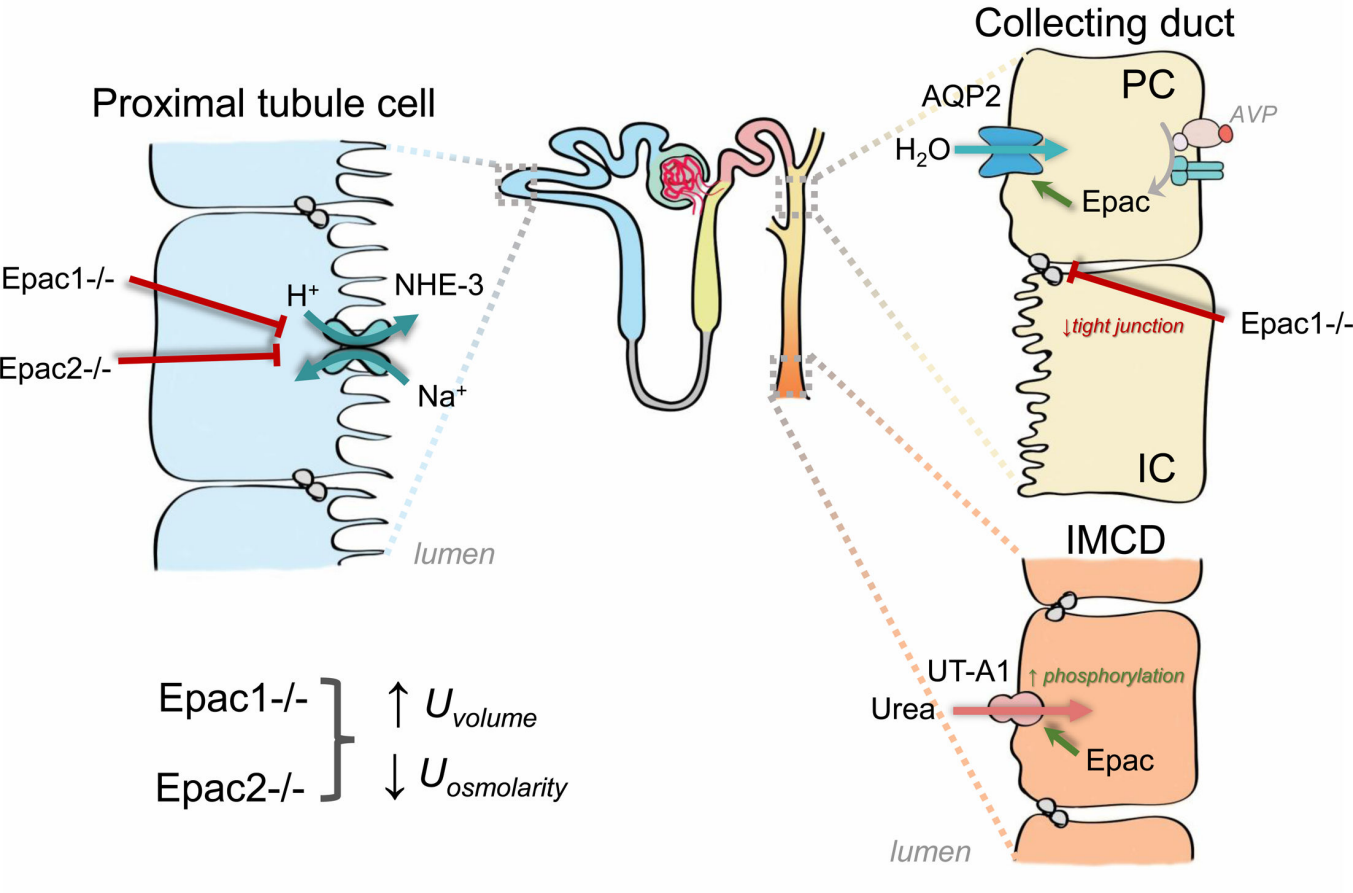
Water balance regulation by Epac1 and Epac2. Epac1 and Epac2 play essential roles in renal water balance by regulating NHE-3, AQP2, and UT-A1. AQP2, aquaporin 2; AVP, arginine vasopressin; IC, intercalated cell; IMCD, inner medullary collecting duct; NHE-3, Na^+^/H^+^ exchanger 3; PC, principal cell, UT-A1, urea transporter A1.

The existing published evidence supports that Epac signaling is a critical regulator of renal water handling (Figure 3). The two different Epac1 knockout mouse models exhibit polyuria at the baseline and have a diminished urinary concentrating ability in response to water deprivation or AVP injection, suggesting a compromised renal water handling [19,32]. AQP2 expression and translocation to the apical membrane is known to play a central role in AVP-controlled water permeability of the collecting duct principal cells to set urinary volume and osmolarity [33,34]. Moreover, selective stimulation of Epac signaling recapitulated AVP-induced intracellular Ca^2+^ oscillations due to release from ryanodine-sensitive endoplasmic reticulum (ER) stores in both perfused inner medullary collecting ducts and cultured collecting duct principal cells. This, in turn, promoted AQP2 trafficking to the apical membrane independently of PKA [35–37]. In addition to regulating AQP2 trafficking, AVP causes sustained transcriptional activation of AQP2 expression in a cAMP/cAMP-responsive regulatory element (CREB)-dependent manner [38–42]. It was further found that PKA inhibitors do not affect AVP-induced AQP2 expression or CREB phosphorylation in cultured mpkCCD_C14_ cells. However, ERK inhibition blocks CREB phosphorylation and reduces AQP2 up-regulation by AVP, indicating an ERK-dependent mechanism [43]. It was later shown that the long-term stimulatory effect of AVP may involve the activation of Epac to augment AQP2 transcription via V2R-cAMP, followed by phosphorylation of the CREB pathway. In contrast, the initial phase requires the involvement of PKA in cultured collecting duct principal cells [44]. Somewhat unexpectedly, AQP2 expression and apical localization were not compromised in mice with deleted Epac1 and Epac2 isoforms, arguing against its central contribution to the polyuric phenotype seen in Epac knockouts [19,32]. The nature of the observed differences between *in vitro* and *in vivo* studies requires careful further investigation.

The accumulation of urea in the inner medulla is an important component of the corticomedullary interstitial osmotic gradient, which determines maximal urinary concentrating ability in the state of anti-diuresis [45]. Urea transporters (UTs) are crucial in urine concentration and fluid balance. UT-A1, expressed in the inner medullary collecting duct, is the primary UT among six known UT-A variants derived from alternative promoters or splicing. AVP increases urea permeability via cAMP by controlling apical localization and phosphorylation of UT-A1 in the inner medulla [46,47]. It was originally thought that AVP stimulates UT-A1 activity in a PKA-dependent manner. However, AVP- and forskolin-induced urea fluxes were only partially inhibited by PKA blockade, suggesting an additional cAMP-dependent but PKA-independent pathway downstream of AVP to augment urea reabsorption [48].

Pharmacological Epac stimulation increased urea reabsorption in the inner medullary collecting ducts by increasing UT-A1 urea transporter phosphorylation via the MEK-ERK pathway, leading to its accumulation on the apical plasma membrane [49]. While PKA phosphorylation sites on UT-A1 have been identified at S486, S499, and S84 [47,50,51], it appears that Epac phosphorylates UT-A1 at a different unidentified site [52]. In addition, physiologically relevant modes of UT-A1 phosphorylation at S494 independent of cAMP-Epac have been reported. While PKC-dependent hypertonicity-stimulated phosphorylation at this site does not promote UT-A1 trafficking to the apical membrane, the S494A mutation reduces UT-A1 membrane abundance, suggesting its role in UT-A1 retention in the plasma membrane [53]. Along these lines, UT-A1 and UT-A3 transporter levels were unchanged upon deletion of Epac1 and Epac2, although their phosphorylation status was not assessed [19,32]. However, wildtype (WT), Epac1^-/-^, and Epac2^-/-^ mice have comparable urea levels in urine at the baseline [19]. In addition, urea-dependent osmotic diuresis was not altered in Epac1^-/-^ mice subjected to a high-protein diet [32]. This argues against defective reabsorption of urea in the inner medullary collecting duct. Nonetheless, Epac isoform deletion led to a significantly increased urea level in response to water deprivation than those seen in WT, which could be, at least partially, due to reduced levels of AVP-dependent UT-A2 levels in the tDL [19]. UT-A2 participates in the medullary urea recycling, with its deletion diminishing urinary concentrating ability [54], thereby recapitulating the phenotype seen in the Epac isoform knockouts [19]. Since previous studies did not detect notable Epac1 and Epac2 levels in the tDL of rodents and humans [16], the down-regulation of UT-A2 levels in Epac isoform knockouts might represent an indirect effect, necessitating future investigation.

As mentioned above, two independent mouse models with Epac1 deletion are consistent with observing a notable impairment of renal water conservation at the baseline and in response to antidiuretic stimuli [19,32]. Using transmission electron microscopy, shorter and less dense tight junctions along the collecting duct were proposed to be causative for the attenuated corticomedullary osmotic gradient and augmented free water clearance [32]. Osmotic diuresis driven by a drastically decreased NHE-3 expression in the PT and diminished urinary concentrating ability in the presence of compensatory elevation of AVP and AQP2 levels in mice lacking Epac1 and Epac2 isoforms was deducted as an underlying mechanism of polyuria in another study [19]. Despite the differences, these models provide strong experimental support for the overall conclusions of the critical role of Epac signaling cascade in governing water handling by the kidney, with both proposed mechanisms likely contributing synergistically to maximize urinary concentrating ability.

## Regulation of solute transport in the renal tubule by Epac

**Figure 4:**
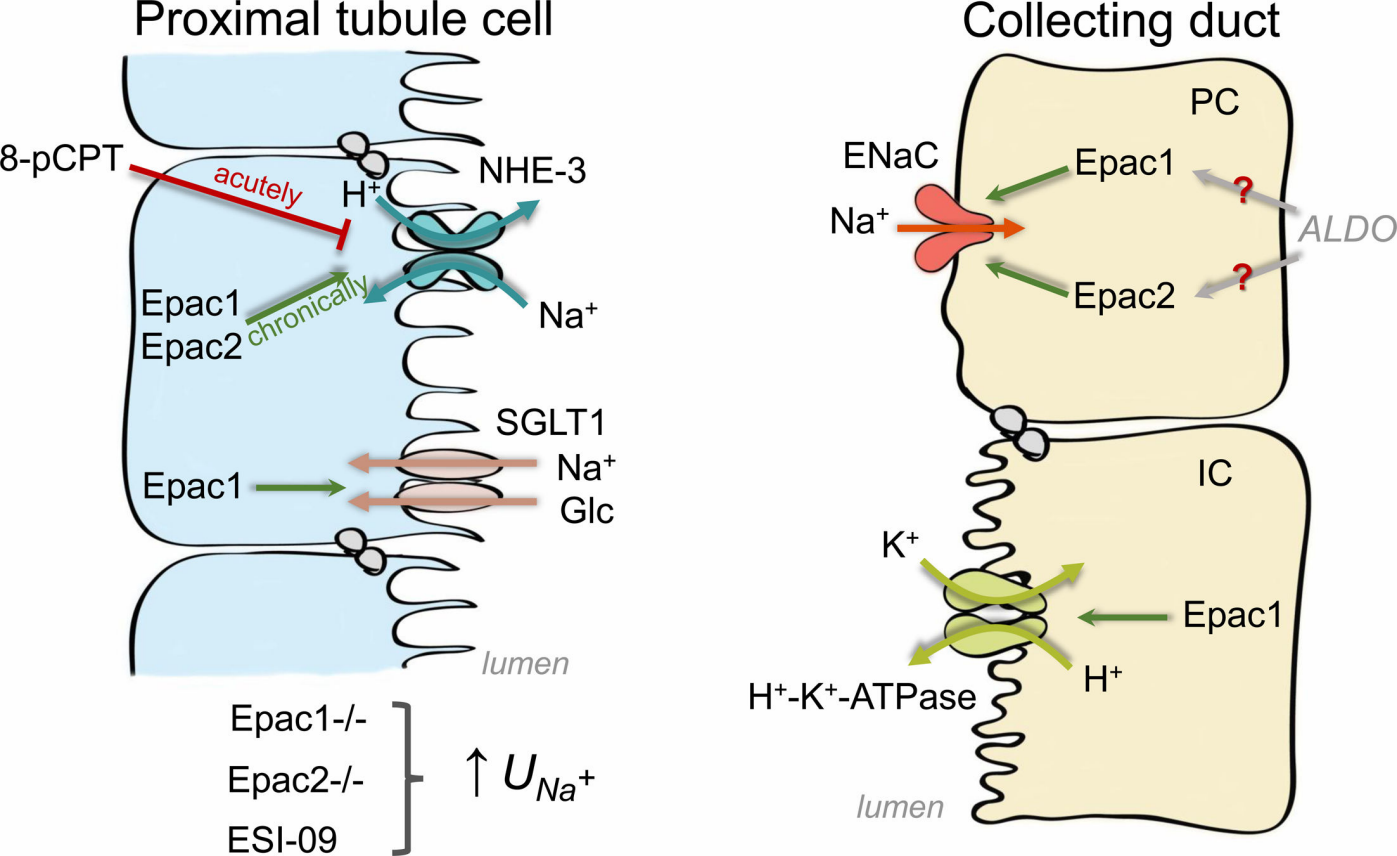
Solute transport regulation by Epac1 and Epac2. PC – principal cell, IC – intercalated cell, NHE-3 – Na^+^/H^+^ exchanger 3, SGLT1 – sodium-glucose cotransporter 1, ENaC – epithelial Na^+^ channel, 8-pCPT – Epac selective cAMP analogue (8-pCPT-2’-O-Me-cAMP), ALDO - aldosterone.

A notable apical localization of Epac isoforms in the renal nephron strongly suggests their involvement in the regulation of solute transport in multiple tubular segments. Indeed, strong but somewhat isolated published evidence demonstrates a critical role of Epac in controlling activity and expression of apical transporters in the PT and the collecting duct system (Figure 4).

### Proximal tubule

NHE-3, a member of the nine isoforms within the mammalian NHE gene family, is predominantly expressed in the PT and to a lesser extent in the thick ascending limb of Henle [55]. It plays a crucial role in sodium reabsorption and maintaining acid–base homeostasis [56]. Second messengers meticulously regulate the function of this specific antiporter [57]. The apical expression of NHE-3 and its localization to the tip of the brush border of PT cells are known to mediate the majority of transcellular Na^+^ reabsorption at this site [58,59]. Intracellular cAMP suppresses NHE-3 activity and reduces sodium absorption in renal and intestinal epithelia, with a family of Na^+^/H^+^ exchanger regulatory factors (NHERF) being essential for cAMP-mediated NHE-3 inhibition [60]. The multi-PDZ domain-containing NHERF1 binds to NHE-3 and transduces the cAMP-mediated inhibitory effect on NHE3 [61–63]. Studies using primary brush border membranes isolated from the kidney cortex of NHERF1^-/-^ mice have demonstrated that NHERF1 is necessary for cAMP-mediated inhibition of NHE3 [64].

cAMP-dependent phosphorylation on the PKA-sensitive S552 site of NHE-3 leads to its myosin VI-dependent translocation to the base (coated pit) region of the brush-border membrane, causing its inactivation [65]. It was further proposed that this mechanism could contribute to reduced fluid reabsorption in the PT during hypervolemic states [58]. Interestingly, the application of Epac selective cAMP analog (8-pCPT-2’-O-Me-cAMP) decreased NHE-3-dependent H^+^ secretion without changes in the total and phosphorylated (pS^552^) NHE-3 levels, whereas the PKA-selective agonist inhibited NHE-3 by increasing pS^552^ NHE-3 [15].

Quite surprisingly, global deletion of either Epac1 or Epac2 led to a drastic decrease in both total and pS^552^ NHE-3 expression levels in the kidney [19]. Of note, the overall renal phenotype of Epac1^-/-^ and Epac2^-/-^ mice was strikingly similar to that seen upon selective ablation of NHE-3 in the PT: moderate polyuria, mild urinary salt wasting, and urinary concentrating defect without apparent alterations in body weight and significant plasma electrolyte levels [66]. Moreover, systemic administration of pan-selective Epac blocker, ESI-09 [67–69], to WT mice induced a concomitant increase in both 24-hour urinary Na^+^ and K^+^ levels, indicative of its significant effect on the inhibition of solute transport in the PT [20]. With regard to the apparent opposite outcomes on NHE-3 function upon *in vitro* pharmacological cAMP-Epac stimulation [15] and *in vivo* genetic Epac deletion [19], it is likely that acute elevations in cAMP levels are instrumental in down-regulating NHE-3 activity via the PKA (and possibly Epac) pathway [70], whereas Epac is necessary for long-term control of NHE-3 expression by stabilizing its interaction with the cytoskeleton via NHERF1 at the brush-border membrane. A similar mechanism involving Epac was shown to regulate plasma membrane levels of cystic fibrosis transmembrane conductance regulator (CFTR) Cl^-^ channels in human airway epithelial cells [71]. It is interesting that parathyroid hormone (PTH) and dopamine increase cAMP levels to inhibit both NHE-3 [72,73] and Na^+^/phosphate cotransporter (NaPi-IIa, *Slc34A1*) in PT cells [74]. However, the cAMP-induced inhibition of NaPi-dependent phosphate transport is PKA-dependent and does not involve Epac [15,75]. This suggests that endocrine factors leading to elevations in intracellular cAMP can target distinct transporting systems in the PT, depending on whether the PKA or Epac pathway is utilized.

In addition to its incretin effect, the gut hormone GLP-1 exhibits natriuretic and diuretic properties [76,77]. Studies on pig kidney epithelial cell lines, LLC-PK1, suggest that GLP-1 probably modulates sodium homeostasis in the kidney by inhibiting NHE-3 activity. Regulation of NHE-3 activity by the GLP-1R agonist exendin-4 is associated with increased NHE-3 phosphorylation without changes in the plasma membrane levels and requires activation of both PKA and Epac. Epac-dependent NHE-3 inhibition involves the MAPK pathway, as pharmacological inhibition of MEK1/2 negates the effect induced by the Epac activator [78]. Angiotensin II (Ang II) promotes bicarbonate absorption in the PT by reducing intracellular cAMP through a Gαi signaling pathway [79]. In LLC-PK1 cells, Ang II decreases Rap1a activation and increases NHE-3 membrane translocation. This effect is reduced with Epac1 and PKA activators or constitutively active Rap1 mutants but increased with a dominant negative Epac1. Ang II also induces inflammatory cytokines (IL-1β, IL-6, IL-8, TNFα) via an Epac1/Rap1-dependent mechanism. NHE-3 inhibitor S3226 or Epac1 and PKA activators can block this cytokine production, revealing an Epac1-Rap1a-NHE-3 pathway important for renal inflammation [80].

The PT is recognized as the only site for reabsorption of filtered glucose via the coordinated actions of the high-capacity and low-affinity Na^+^/glucose cotransporter 2 (SGLT2) and low-capacity and high-affinity Na^+^/glucose cotransporter 1 (SGLT1) to prevent excretion of glucose in urine [59,81]. Glucose filtered in the glomerulus is reabsorbed by SGLTs in renal PT cells, regulated by various signals that increase intracellular cAMP [82]. It was shown that cAMP-dependent stimulation of PKA and Epac pathways increased expression and membrane lipid raft accumulation of SGLT1 in PT cells to augment α-methyl-D-glucopyranoside (α-MG) uptake via mechanisms that involved ERK/MAPK, and the nuclear factor kappa B (NF-κB) and F-actin organization, respectively [83]. In recent years, SGLT2 blockers, such as canagliflozin, dapagliflozin, and empagliflozin, have been proven to be highly beneficial in the treatment of type 2 diabetes by reducing plasma glucose levels and reducing the risk of cardiovascular complications [84–86]. Of note, SGLT2 inhibition induces a robust up-regulation of SGLT1-mediated glucose uptake in the PT [81,87]. This could imply a role of the Epac signaling cascade in controlling glucose transport, particularly in diabetic states. However, there is no published evidence in support of this hypothesis.

### Collecting duct

The collecting duct is known to play a physiologically relevant role in fine-tuning Na^+^ reabsorption in response to variations in dietary salt intake to maintain systemic Na^+^ homeostasis and set the baseline of blood pressure [88–90]. Activity of the aldosterone-sensitive epithelial Na^+^ channel (ENaC) localized to the apical plasma membrane of principal cells is the rate-limiting step in Na^+^ reabsorption in the collecting duct [91]. Loss-of-function or gain-of-function mutations in ENaC lead to monogenic forms of hypotension or hypertension [92–94]. Moreover, numerous ENaC polymorphisms are associated with salt-sensitivity of blood pressure in the clinical setting [95–97]. Abundant published evidence demonstrates that elevations of intracellular cAMP levels increase apical localization and ENaC activity in both *in vitro* and *ex vivo* settings [98–100], arguing for a potential involvement of Epac-dependent signaling cascades. Indeed, acute application of pan-selective (ESI-09) and Epac2-specific (ESI-05) antagonists significantly decreased ENaC activity in WT but not in Epac-deficient mice. Importantly, the magnitude of inhibition was higher when animals were preconditioned with Na^+^ deficient diet, pointing to Epac as a critical downstream target of aldosterone-mediated activation of ENaC. Epac1^-/-^ and Epac2^-/-^ mice consistently have lower ENaC activity in split-opened collecting ducts and develop urinary Na^+^ wasting when challenged with a Na^+^-deficient diet. Interestingly, concomitant Epac1 and Epac2 deletion produced a slightly stronger phenotype, but this was achieved at the expense of significantly higher aldosterone levels, suggesting similar but nonredundant actions of Epac1 and Epac2 in stimulation of ENaC activity during variations in dietary salt intake [20]. This indicates that Epac blockers could be used as anti-hypertensive and natriuretic agents in various pathological states associated with elevated ENaC activity. Future studies need to be performed to test this possibility.

Intercalated cells of the collecting duct are generally recognized for playing a central role in the regulation of systemic acid-base homeostasis by secreting either H^+^ (A-type) or HCO_3_^-^ (B-type) to maintain physiological pH [22,101]. H^+^-K^+^-ATPases are ion pumps that use energy from ATP hydrolysis to transport H^+^ in exchange for K^+^ against their concentration gradients [102]. In the kidney, H^+^-K^+^ ATPase has been shown to be predominantly expressed on the apical membrane of intercalated cells of both types, with its activity and expression being stimulated by dietary K^+^ deficiency and acidosis [103]. More specifically, two H^+^-K^+^ ATPase isoforms having different catalytic α subunits (α_1_ or gastric and α_2_ or non-gastric/colonic) have been present in the intercalated cells, and mice lacking either isoform exhibit notable deficiencies in maintaining potassium and acid–base balance [104]. It was shown that several endocrine factors augment H^+^-K^+^ ATPase activity by stimulation of cAMP/Epac1/Rap-1/Raf-B/ERK cascade in rat collecting duct, and this regulation was independent of PKA [14]. Calcitonin and isoproterenol activate H^+^-K^+^-ATPase in rat cortical collecting ducts via cAMP, but only the effect of isoproterenol is sensitive to PKA inhibition. Anti-Epac1 antibodies block calcitonin-, but not isoproterenol-induced activation of the pump. Calcitonin activates H^+^-K^+^-ATPase with PKA-independent ERK activation, suppressed by U0126 or antibodies against Rap1 or Raf-B, but not Ras or Raf-1. These results indicate that A- and B-intercalated cells could implement different cAMP pathways, cAMP/Epac1/Rap1/Raf-B/ERK or cAMP/PKA, to regulate H^+^-K^+^-ATPase activity [49]. It is tempting to speculate that Epac signaling could be essential in controlling acid–base balance by not only regulating H^+^ secretion in the collecting duct but also HCO_3_^-^ reabsorption in the PT by controlling NHE-3 expression and activity, as was discussed above. Yet, the direct evidence in support of this concept is currently missing.

## Role of Epac signaling in renal pathologies

As a key stress-response pathway, altered or deficient Epac signaling has been found during the development of both acute and chronic renal disorders.

### Acute kidney injury (AKI)

AKI is a common clinical pathology, which is generally associated with an abrupt and sustained decrease in renal function (glomerular filtration rate, GFR), posing a substantial risk for the development of end-stage renal disease (ESRD) and is associated with high rates of morbidity and mortality [105]. A decrease in renal perfusion due to excessive vasoconstriction, blood volume loss, and cardiac failure causes ischemia, which is one of the most common causes of AKI [106]. The ischemia followed by reperfusion (ischemia-reperfusion injury or IRI) is generally associated with overproduction of reactive oxygen species (ROS) and formation of pro-inflammatory cytokines, such as tumor necrosis factor (TNF)-α and interleukin (IL)-6 causing injury of renal nephron epithelial cells, particularly the PT (loss of apical brush border and tubular integrity due to cell detachment) having a very high metabolic rate [106,107]. Selective stimulation of Epac signaling with intrarenal administration of 8-pCPT-2′-O-Me-cAMP reduced renal failure (less plasma urea) and led to improvement of tubular barrier function (decreased expression of the tubular cell stress marker clusterin-α, and lateral expression of β-catenin after ischemia) [17]. It was further suggested that pharmacological stimulation of Epac (most likely Epac1 isoform) reduced superoxide production by mitochondria of PT cells, thereby ameliorating the IRI-induced oxidative stress [108]. Consistently, the activation of cAMP-Epac signaling in response to inhibition of purinergic P2Y_12_ receptor with ticagrelor improved renal function (lower creatinine and BUN) and ameliorated structural damage in a rat model of IRI, and this was reversed upon application of a selective Epac1 blocker, R-CE3F4 [109]. Moreover, anti-cancer chemotherapy agents, most commonly cisplatin and doxorubicin, exhibit notable adverse nephrotoxicity primarily via overproduction of ROS [110] to drive PT cell apoptosis and fibrosis. The activation of Epac but not PKA protected against apoptosis of murine PT cells upon cisplatin treatment, and these beneficial effects were abolished upon silencing of Epac1 expression [111].

Endothelial progenitor cells (EPCs) can migrate into post-ischemic kidneys, protecting them from acute ischemic damage [112]. EPACs have been shown to play beneficial roles in different ischemic conditions, including myocardial and renal. Pre-activation of EPACs with an activator of Epac increases their protective potency during IRI [113].

Overall, the available experimental evidence does not directly couple dysregulated Epac signaling with the severity of renal damage during AKI. However, it points to beneficial renoprotective effects of a selective Epac (specifically Epac1) activation to reduce ROS levels and preserve tubular integrity. The translational component of the implementation of Epac-dependent signaling in AKI and other related renal pathologies is expected to be promising.

### Chronic kidney disease (CKD)

CKD is a broad spectrum of diseases of different etiology persisting over 3 months (90 days), associated with abnormalities in kidney morphology/structure and causing a substantial (50% or more) reduction in GFR [114]. The most common driving factors for CKD progression include hypertension, diabetes mellitus (hyperglycemia), and immune system activation/inflammation [115]. Cyclic AMP plays a role in renal physiology, and abnormal cAMP signaling is linked to kidney fibrosis and renal failure [116]. The altered architecture of glomeruli, particularly podocyte damage/injury, is one of the hallmarks of CKD, leading to a compromised filtration barrier and proteinuria [117]. It was recently shown that the deletion of Epac1 globally and specifically in podocytes accelerates the progression of glomerular nephritis induced by a nephrotoxic serum. Moreover, the stimulation of Epac signaling with 8-pCPT-2′-O-Me-cAMP attenuated the progression of the disease by improving kidney function, decreasing structural damage, and reducing inflammation. At the cellular level, it was demonstrated that Epac1 signaling induces a Warburg-like metabolic reprogramming in podocytes to augment glycolysis and lactate production, resulting in improved podocyte viability [118]. Somewhat unexpectedly, the stimulation of PKA- but not Epac-dependent signaling was shown to mediate the protective effects of cAMP in podocytes to reduce albuminuria in adriamycin and puromycin aminonucleoside-induced nephrosis model [119,120]. However, the stimulation of Epac was shown to prevent down-regulation of ezrin/radixin/moesin phosphorylation in these experimental models [120]. Besides differences in experimental models and conditions, cAMP signaling appears to be highly compartmentalized in glomerular cells, triggering distinct downstream effectors. Thus, cAMP-dependent activation of TRPC6, the Ca^2+^-permeable channel implicated in late onset of the focal segmental glomerulosclerosis [121,122], does not involve either PKA or Epac [123]. In contrast with its role in podocytes, the activation of Epac signaling could be detrimental in glomerular smooth muscle origin mesangial cells, which can contract to regulate blood flow across the glomerular capillaries. Ang II has been shown to stimulate AT1R in mesangial cells to increase extracellular deposition of collagens to promote glomerulosclerosis [124]. It appears that downstream activation of phosphoinositide 3-kinase (PI3K) to drive collagen synthesis required trans-activation of epidermal growth factor (EGF) receptor in an Epac- but not a PKA-dependent manner [125,126]. One study indicates that Epac activates PI3K/Akt signaling via the Src kinase and EGFR pathway [125], while another report suggests that TGF_β_RI transactivation mediates this pathway [126].

Interstitial fibrosis is another hallmark of CKD and particularly diabetic kidney disease [127]. Sustained activation of the renin–angiotensin system is associated with the progression of CKD, such as diabetic nephropathy, which accounts for over 30% of end-stage renal diseases, in part by Ang II-induced activation of NADPH oxidase and production of ROS and inflammatory cytokines. Adiponectin, the hormone known to improve insulin sensitivity and reduce inflammation [128], interacted with its adiponectin receptor type 1 to prevent Ang II-dependent activation of NADPH oxidase and NF-κB activation, thereby exhibiting renoprotective actions via AMPK- and Epac- but not via PKA-dependent pathways [129]. Ang II was also shown to increase the production of inflammatory cytokines, including IL-1β, IL-6, and TNF-β, which was reduced in response to stimulation of Epac-Rap1a signaling [80]. Thus, it is proposed that Epac signaling could be instrumental in diminishing tubulointerstitial inflammation in CKD to reduce fibrosis. Along these lines, it was suggested that cAMP levels are reduced in fibrotic kidney tissues due to up-regulation of phosphodiesterase type 4 (PDE4). Inhibition or silencing of PDE4 restored cAMP levels, leading to stimulation of Epac1-Rap1 signaling to ameliorate extracellular matrix deposition [130]. Moreover, the administration of the Epac agonist 8-pCPT-2'-O-Me-cAMP to diabetic *db/db* mice led to the inhibition of tubulointerstitial inflammation (reduced infiltration of macrophages) to alleviate interstitial fibrosis by inhibiting p-STAT3, MCP-1, IL-6, and TNF-α expression in the renal cortex [131]. On the contrary, high glucose levels in diabetic mice (induced by STZ injections) increased transcription and translation of Epac1, leading to Akt phosphorylation and modulation of cell cycle events, which are independent of PKA, culminating in the cellular hypertrophy of PT cells during the development of diabetic nephropathy [132]. Hypertrophy of PT cells is one of the drivers leading to interstitial fibrosis. The nature of these discrepancies is not clear.

### Polycystic kidney disease (PKD)

PKD is one of the most common hereditary disorders (prevalence 1:600–1000), which is characterized by the formation and subsequent enlargement of fluid-filled cysts in renal parenchyma, causing a gradual decline in renal function and eventually progressing to ESRD [133,134]. In addition, extrarenal PKD manifestations include cyst formation in other organs, most commonly the liver and pancreas, and intracranial and vascular aneurysms [135]. Compelling experimental evidence demonstrates the central role of increased intracellular cAMP levels in driving cystogenesis by stimulating luminal Cl^-^ secretion, augmenting the proliferation of cyst cells, and inducing interstitial fibrosis [136–139]. To date, the only FDA-approved PKD treatment, V2R antagonist tolvaptan, was shown to decrease cAMP levels, thereby delaying PKD progression, while it exerts deleterious effects on renal water conservation and has notable liver toxicity [140]. Interestingly, cAMP-driven cystogenesis seems exclusively PKA-dependent but not Epac-dependent in the renal epithelia. PKA-I activity directly correlated with the rate of cystogenesis in homologous autosomal dominant PKD mouse models, whereas Epac activation had little effect [141]. Consistently, the inhibition of PKA with H89 blocked *in vitro* proliferation of primary cultured cyst cells from PKD patients by precluding ERK1/2 activation [142]. While this suggests targeting PKA rather than Epac as a potential new avenue in the treatment of PKD [143], both PKA and Epac were involved in promoting cAMP-stimulated growth of cholangiocyte-derived liver cysts via the MEK-ERK1/2 cascade in a rat homologous model of autosomal recessive PKD [144].

## Conclusions and future perspectives

In summary, extensive research indicates that Epac signaling pathways contribute significantly to regulating cAMP-mediated functions within the renal system and play essential roles in the pathogenesis of kidney diseases. However, it is important to note that Epac signaling does not influence all cAMP functions in the kidney. For instance, the kidney regulates K^+^ homeostasis by modulating K^+^ secretory channels like ROMK in the collecting duct system. Ang II inhibits ROMK activity through AT1R and the PLC/PKC pathway under low dietary K^+^ but activates it via AT2R through a NO/cGMP pathway involving cAMP/PKA-dependent signaling independently of Epac in high potassium fed rats [145,146].

While cAMP acts through various effectors like Epac and PKA, dissecting the spatial and temporal contributions of Epac and PKA in the kidney is challenging. Current knowledge mostly comes from *in vitro* cell culture studies. Identifying each cAMP effector’s role in living organisms using genetic or pharmacological methods is still difficult. Schwärzel and colleagues have used an innovative approach with transgenic photoactive AC bPAC to generate cAMP pulses in specific cell types. This optogenetic method, combined with manipulations of PKA or Epac, helps explore their roles in Drosophila Malpighian tubules' principal and stellate cells. Their findings reveal that Epac is crucial for stimulated fluid secretion in both cell types, while PKA is essential for basal secretion in principal cells and stimulated secretion in stellate cells [147]. This study shows that optogenetics is a promising approach for dissecting cAMP signaling *in vivo*. Extending similar methods to higher organisms could significantly improve our understanding of cAMP signaling with precise spatial and temporal detail.

Within the Epac family, the kidney presents a unique organ where the expression of Epac1 and Epac2 is abundant and largely overlapping, and both isoforms seem to exert similar functions, suggesting that they are likely involved in regulating a broad spectrum of cellular processes. It remains unclear whether their roles are additive, synergistic, or isoform-dependent for each particular case. Moreover, as intracellular mediators of cAMP downstream of various stress hormones, renal phenotypes in mice with global Epac knockout might also, at least partially, result from extra-renal Epac ablation. A careful examination of the aforementioned physiological and pathophysiological processes in mice with kidney- and even renal tubule segment-specific deletion of individual or both Epac isoforms are needed to clarify this.

Accumulating evidence supports the view of dysregulated Epac signaling in both acute and chronic renal pathologies. However, significant challenges remain in translating these findings into clinical applications. Primarily, there is a lack of relevant data derived from human samples. Future research utilizing human kidney tissue, organoids, and induced pluripotent stem cells (iPSCs) is essential to elucidate the putative roles and pharmacological potential of Epac cascade in the clinical setting. Although human kidney tissues provide the most accurate representation of human kidney biology and disease due to their intact complex architecture and cellular interactions, and can directly correlate findings with clinical outcomes, obtaining human kidney tissues is challenging due to limited availability and ethical considerations. Additionally, manipulating these tissues genetically or pharmacologically is challenging, and sample variability may influence consistency. Recent advances in organoid culture development offer a reasonable alternative, since organoids replicate the 3D architecture of the kidney, providing a more realistic model when compared with the traditional 2D cell cultures. Additionally, organoids can be genetically modified to investigate specific genes and pathways involved in kidney diseases and can be used to model various kidney conditions and test drug responses in a controlled environment [148,149]. Having said that, organoids cannot fully replicate the complexity and intricate interactions and functions of a mature kidney. Variability in organoid formation and function may also influence reproducibility. Finally, iPSCs, which can differentiate into any cell type including kidney cells, are highly versatile for studying kidney biology. iPSCs derived from patients with specific kidney diseases are particularly valuable for personalized disease modeling and drug testing [150,151]. However, like organoids, creating sophisticated models that accurately mimic human kidney behavior using iPSCs remains challenging. The efficiency of reprogramming somatic cells into iPSCs varies, potentially affecting result consistency. Additionally, iPSCs may acquire genetic mutations during reprogramming and culture, affecting their reliability. Achieving complete and accurate differentiation into kidney cells also presents technical difficulties.

The role of Epac signaling in kidney function remains unclear due to mixed experimental results. While some studies show that genetic deletion or inhibition of Epac ameliorates pathophysiological manifestations, other studies report beneficial actions upon Epac activation. Interestingly, mice with concomitant deletion of both Epac1 and Epac2 do not exhibit developmental defects and have grossly normal phenotypes at baseline. This indicates that targeting Epac signaling would likely be well tolerated with respect to toxicity and adverse effects if highly selective modulators are developed.

The development of small molecule Epac-specific modulators represents a significant milestone in the cAMP/Epac signaling field. These Epac activators and inhibitors serve as invaluable pharmacological tools for dissecting Epac-specific cellular signaling pathways and for evaluating the therapeutic potential of targeting Epac in various preclinical disease models. Numerous comprehensive reviews have been published on this topic [152–157]. Although both Epac antagonists and agonists have shown promise in animal models [12,20,155,156], further optimization and preclinical evaluation are needed to develop potent, specific, and safe drug candidates. Future research should address these gaps to understand better the multifaceted roles of Epac1 and Epac2 in kidney function and guide the development of precision therapeutic interventions for renal and potentially cardiorenal diseases.

## Abbreviations

AKI: acute kidney injury
AQP: aquaporin
AVP: arginine vasopressin
Ang II: angiotensin II
CCD: cortical collecting ducts
CFTR: cystic fibrosis transmembrane conductance regulator
CKD: chronic kidney disease
EGF: epidermal growth factor
ENaC: epithelial Na^+^ channel
EPCs: endothelial progenitor cells
EPK: extracellular signal-regulated kinase
ESRD: end-stage renal disease
Epac: exchange proteins directly activated by cAMP
GFR: glomerular filtration rate
IL: interleukin
IRI: ischemia-reperfusion injury
NCC: Na^+^-Cl^-^ cotransporter
NHE-3: Na^+^/H^+^ exchanger type 3
NHERF: Na^+^/H^+^ exchanger regulatory factors
NKCC2: Na^+^, K^+^, and Cl^-^ transporter
PI3K: phosphoinositide 3-kinase
PKA: protein kinase A
PKD: polycystic kidney disease
PT: proximal tubule
PTH: parathyroid hormone
ROS: reactive oxygen species
TAL: thick ascending limbs of Henle's loop
TNF: tumor necrosis factor
UT: urea transporters
V2R: vasopressin receptors type 2
cAMP: 3',5'-cyclic adenosine monophosphate
tAL: thin ascending limbs of Henle's loop
tDL: thin descending limbs of Henle's loop
